# Comparison of a one-time educational intervention to a teach-to-goal educational intervention for self-management of heart failure: design of a randomized controlled trial

**DOI:** 10.1186/1472-6963-9-99

**Published:** 2009-06-11

**Authors:** Darren A DeWalt, Kimberly A Broucksou, Victoria Hawk, David W Baker, Dean Schillinger, Bernice Ruo, Kirsten Bibbins-Domingo, Mark Holmes, Morris Weinberger, Aurelia Macabasco-O'Connell, Michael Pignone

**Affiliations:** 1 Cecil G Sheps Center for Health Services Research, Division of General Internal Medicine, University of North Carolina at Chapel Hill, Chapel Hill, NC, USA; 2 Health Policy and Management, University of North Carolina at Chapel Hill, Chapel Hill, NC, USA; 3 Feinberg School of Medicine, Northwestern University, Chicago, Ill, USA; 4 Center for Vulnerable Populations, Department of Medicine, San Francisco General Hospital, University of California San Francisco, San Francisco, CA, USA; 5 Olive View – UCLA Medical Center, UCLA School of Nursing, University of California Los Angeles, Los Angeles, CA, USA

## Abstract

**Background:**

Heart failure (HF) is common, costly and associated with significant morbidity and poor quality of life, particularly for patients with low socioeconomic status. Self-management training has been shown to reduce HF related morbidity and hospitalization rates, but there is uncertainty about how best to deliver such training and what patients benefit. This study compares a single session self-management HF training program against a multiple session training intervention and examines whether their effects differ by literacy level.

**Methods/Design:**

In this randomized controlled multi-site trial, English and Spanish-speaking patients are recruited from university-affiliated General Internal Medicine and Cardiology clinics at 4 sites across the United States. Eligible patients have HF with New York Heart Association class II-IV symptoms and are prescribed a loop diuretic. Baseline data, including literacy level, are collected at enrollment and follow-up surveys are conducted at 1, 6 and 12 months

Upon enrollment, both the control and intervention groups receive the same 40 minute, literacy-sensitive, in-person, HF education session covering the 4 key self-management components of daily self assessment and having a plan, salt avoidance, exercise, and medication adherence. All participants also receive a literacy-sensitive workbook and a digital bathroom scale. After the baseline education was completed, patients are randomly allocated to return to usual care or to receive ongoing education and training. The intervention group receives an additional 20 minutes of education on weight and symptom-based diuretic self-adjustment, as well as periodic follow-up phone calls from the educator over the course of 1 year. These phone calls are designed to reinforce the education, assess participant knowledge of the education and address barriers to success.

The primary outcome is the combined incidence of all cause hospitalization and death. Secondary outcomes include HF-related quality of life, HF-related hospitalizations, knowledge regarding HF, self-care behavior, and self-efficacy. The effects of each intervention will be stratified by patient literacy, in order to identify any differential effects.

**Discussion:**

Enrollment of the proposed 660 subjects will continue through the end of 2009. Outcome assessments are projected to be completed by early 2011.

**Trial Registration:**

ClinicalTrials.gov  NCT00378950

## Background

Heart failure (HF) is common, costly, and associated with significant mortality, morbidity and poor quality of life. HF affects 5 million people in the US, causing 266,000 deaths and costing 25.8 billion dollars annually. [1] Research has shown that a combination of optimal medical care and careful self-management can reduce HF-related morbidity and mortality. [2] Optimal HF treatment, therefore, includes prescription of effective medications (e.g., ACE inhibitors and beta-blockers for systolic HF) and appropriate diagnostic testing (e.g., echocardiography), as well as the use of care management interventions, including self-management training. Self-management training teaches key self-care skills and reinforces behaviors, including symptom recognition, weight monitoring, dietary salt restriction, exercise, medication adherence, and a plan for what to do in the event of a HF exacerbation.

McAlister and colleagues performed a systematic review of 29 trials examining HF disease management programs, including 4 trials that focused on teaching patients self-management skills. While they found self-care training to be effective in reducing hospitalizations, national studies have shown that only a small percentage of practices and health systems provide HF self-management support. Significant uncertainty remains about how to deliver HF self-management training to ensure success, which populations benefit most from these programs, and how feasible these programs are. This uncertainty has contributed to the gap in translating findings from HF research into practice.

Literacy is an important patient characteristic that may influence the patient's ability to benefit from education interventions, including heart failure self-management training programs. Low or inadequate literacy is common (40% of the adult population in some studies) and is associated with multiple adverse health outcomes, including heart failure, all-cause hospitalization, and mortality. [3-5] Wolf and colleagues demonstrated an association between low literacy and the diagnosis of heart failure among Medicare managed care enrollees. [6] Prior research has shown that standard care management interventions often fail to reach populations with limited literacy. As such, interventions specifically designed to meet the needs of patients with low literacy and heart failure could have particular public health relevance.

### A. Pilot Research

Our research team has developed and tested an intervention to improve self-management among patients with HF and low literacy. [7,8] In this single-site trial, 123 patients with HF were randomly assigned to receive a multifaceted HF self-management training intervention verses a HF educational brochure (written at the 7^th ^grade level), each as adjuncts to usual care from their provider. The intervention included an initial one-hour educational session with a trained health educator, a digital bathroom scale, an educational notebook, and a series of 6 follow-up telephone calls over 2 months followed by monthly calls for 10 months to reinforce the educational messages. The intervention increased HF-related knowledge (+12%), self-reported daily weight measurement (88% vs. 21%), and HF-related self-efficacy, but did not affect HF-related quality of life. [8] Overall, the intervention reduced the combined endpoint of hospitalization or death by 44% (IRR 0.56, 95% CI 0.32, 0.95). While the sample size of this study did not provide sufficient power to make a definitive determination, participants with low literacy appeared to benefit as much and more than those with adequate literacy. The study's main limitations were that it was conducted at a single center, used non-blinded outcome assessment, and required statistical adjustment to control for baseline differences in confounding variables. In addition, it was not clear how much of the multifaceted intervention was necessary for success. The present study seeks to extend the results of our single-site pilot study.

### B. Aims

The main objectives of the current study are: 1) to determine whether a literacy-sensitive, multi-sessioneducational interventionthat teachespatients HF self-careskills until they reachbehavioral goals (Teach to Goal – TTG)is superior to a single session Brief Educational Intervention (BEI)for the combined outcome of incidence of hospitalization or death over 1 year, and 2) to determine the size of the effect in patients with lower literacy skills compared with higher literacy skills. The secondary aim is to determine if the TTG is more effective than the BEI for the outcome of HF-related quality of life and adoption of appropriate self-care knowledge, and behaviors, and if the difference in the primary outcome of hospitalization or death are mediated through changes in knowledge, self-efficacy, or improved self-care behaviors.

## Methods/Design

### A. Overview

The study was designed as a randomized controlled trial of 660 participants, with enrollment starting in early 2007, at three sites: University of North Carolina Heart Failure and General Internal Medicine Clinics, Northwestern Medical Faculty Foundation Heart Failure and General Internal Medicine Clinics and University of California San Francisco's Cardiology and Internal Medicine Clinics at San Francisco General Hospital. A fourth site at Olive View-UCLA Medical Center was added and began recruitment of participants in early 2009. At each site, baseline data are collected in person and via electronic record at enrollment into the program. Follow-up data collections at 1 month, 6 months and 12 months are preformed by phone interview by an independent survey organization blinded to intervention status. Approval was granted from the office of Human Research Ethics at the University of North Carolina to conduct this multi site study as well as from each sites governing IRB. We also convened a Data Safety and Monitoring Board, which meets annually to ensure the safety of the intervention and provide advice on study-related issues.

### B. Identifying potential participants

Lists of potential participants are obtained from clinical and billing databases, daily schedules, and from physicians within the designated clinics at the study sites. The research assistants (RAs) then examine the potential participants' medical records for the eligibility criteria as listed in Table 1. If the electronic medical record contains information supporting that the patient meets eligibility, and the patient's physician agrees to the patient participating in the study, the RA then meets with the patient, confirms some of the eligibility requirements and performs a mini cognition screener [9]. The patient is then given details about the participants' obligations in the study and informed that each participant would receive $100 in gift cards over the course of the year long study. If the patient agrees to participate, the RA then confers with the physician and confirms a target weight, a diuretic adjustment schedule and other information that will be needed for the enrollment.

**Table 1:** Eligibility Criteria

**Inclusion Criteria**	**Exclusion Criteria**
1. Diagnosis of HF	1. Inadequate vision (can not see materials)
2. At least 1 of the following	2. On dialysis or starting it within a year
a. LVH on ECG or echocardiogram	3. Severe valvular disease
b. Ejection fraction less than 50%	4. Using oxygen for COPD
c. Pulmonary edema on CXR	5. Life expectancy less than 1 year
d. Elevated B-type Natiuretic Peptide	6. Unable to pass a mini cog cognitive screener – recall 1 of three words and clock drawing [9]
3. A loop diuretic	7. Lives in a nursing facility or other situation where they do not have control of medication
4. NYHA Class II symptoms or higher	
5. Age 20 or older	
6. Has a working phone	
7. Speaks English of Spanish (Spanish Speaking patients recruited at UCSF and UCLA only)	

The RA keeps a log of all patient records reviewed with demographic information including age, race, ethnicity, whether the patient was potentially eligible, and whether he or she agreed to participate. These data provide a breakdown of the overall pool of patients we have accessed and the ability to compare enrolled versus non-enrolled patients so as to assess representativeness.

### C. Initial enrollment- baseline questionnaire

**Figure 1:**
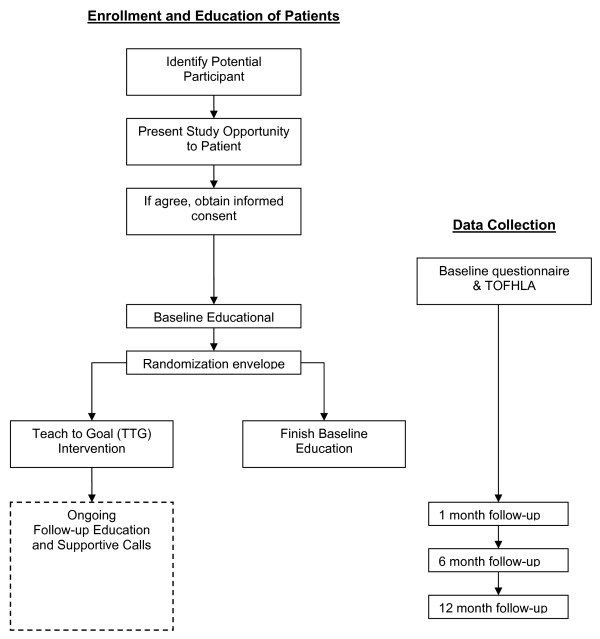
**Study flow chart**.

Once an eligible patient has agreed to participate, the RA arranges the initial enrollment session. The RA obtains informed consent, collects contact information, administers the baseline questionnaire, and assesses literacy status using the short Test of Functional Health Literacy in Adults (s-TOFHLA) (See Figure 1 – Flow Diagram). [10] Immediately after the s-TOFHLA is administered, the RA scores it as either adequate (>= 23 out of 36) or inadequate/marginal (below 23 out of 36). The results of the literacy screening are then used to determine which randomization envelope to pull.

### D. Both Groups – Brief Education Intervention (BEI)

The next step, which is performed directly following the collection of the baseline data or within 2 weeks, is for the patient to meet with the health educator and receive the BEI session. All patients in the study (both control and intervention groups) receive the basic heart failure educational session. Although this level of training exceeds the usual level of care in most settings, we felt it was necessary to include it as our control condition (minimum baseline that all patients should receive) based on the best available evidence and practice guidelines. The initial educational session is approximately 40 minutes long and includes review of the following four HF topics: daily self assessment and having a plan, salt avoidance, exercise, medication adherence (See Table 2). The patient also receives the *Caring for Your Heart: Living Well with Heart Failure *educational manual , [11] which is used to guide these sessions, and a new digital bathroom scale.

**Table 2:** In person training session outline

**In Person Training Session**
**BEI Session**

• ***Overview of Heart Failure***

• ***Medication Adherence***
◦ Taking pills at right times/not skipping doses
◦ Instruction on refilling prescriptions
◦ Discussing systems for taking pills – pill chart
◦ Stress bringing pill bottles to every doctor's visit
◦ Instruction on identifying the water pill

• ***Salt Avoidance***
◦ How salt effects the body
◦ Most foods contain salt
◦ Tips to decrease salt
◦ Common food high and low in salt
◦ How to read food labels
◦ Eating food with 140 mg/serving or less

• ***Exercise ***(instruct only if approved by patient's MD)
◦ Benefits of exercise
◦ Is patient exercising now?
◦ Start slowly, work up to more
◦ Signs for when to stop exercising

• ***Daily Self Assessment***
◦ Assessing shortness of breath when walking
◦ Assess shortness of breath when sleeping
◦ Assessing dizziness/faintness
◦ Assessing swelling of the legs
◦ Weighting self daily
◦ Know target weight

**(RANDOMIZATION TAKES PLACE)**

• ***BEI Conclusion – Having a Plan***
◦ Review the 4 sections
◦ Review when to call the doctor
◦ Write doctor's phone number in workbook
◦ Review scale and how to use it
◦ Call your doctor with any questions

**TTG Session**

• ***Diuretic Self Adjustment & Having a Plan***
◦ Set target weight and record on Water Pill Guide
◦ Set diuretic adjustment schedule and record on Water Pill Guide
◦ Identifying water pill with sticker on bottle
◦ Explain how to record daily weights and doses
◦ Mailing weight and dose logs back to educator
◦ Practice diuretic adjustment with examples of different weight values
◦ When to call the doctor

• ***TTG Follow-up***
◦ Set up Follow-up phone calls
◦ Review recordkeeping and sending in logs to educator

Each site has a health educator who conducts the initial education sessions and the follow-up phone calls for the intervention group. Two of the educators are registered dieticians, both having experience counseling patients in clinical settings. The other 2 have bachelor's degrees and have worked as health educators with clients regarding sex education, disease, and pregnancy prevention. None of the educators had any specific experience or training in HF before this study. To develop and standardize our educational protocol, the three original site educators and the study investigators convened for a one day training prior to beginning enrollment. Following that meeting, one investigator conducted weekly calls with the educators to further develop the educational protocol and to ensure that the delivery of the education was similar across all three sites. The educator at UCLA received training at the UNC site and extensive follow-up over the phone. In addition, sample education calls between the patient and the educator were taped during the initial phases of the study for feedback and quality assurance.

### E. Randomization

Allocation to study group is done with concealed, stratified, block randomization by the statistical team at UNC. Randomization is stratified by site, language (Spanish or English) and literacy level to ensure equal distribution of intervention and control patients. We randomized patients in blocks of variable size to minimize the effect of time on the distribution of patients between intervention and control. Randomization assignments are placed in sets of opaque envelopes and distributed to the health educators at each site. After literacy status is determined and the BEI is delivered, the health educator opens the opaque envelope and learns the intervention status of the patient. If the patient is randomized into the control arm, the education session is concluded with a brief summary of the information discussed and the patient has no further contact with the educator throughout the rest of the study. If the patient is randomized into the intervention arm, the educator proceeds into the additional section of the education for that group.

### F. Intervention Group-Teach to Goal (TTG) Education

The additional education given to the TTG group consists of more specific instruction on daily weight monitoring and recording, and instruction on diuretic self-adjustment. Over the next 12 months the patient receives several follow-up phone calls from the educator to reinforce the education and to guide the patient toward better self-management skills.

**Figure 2:**
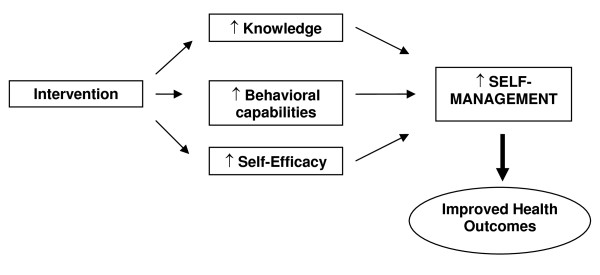
**Conceptual model of the intervention to improve HF self-management**.

The TTG protocol was developed for this study based on previous research and represents an overt effort on our part to design a method for integrating complex self-management into daily life. [7,8] It is based on social cognitive theory because of its strong empiric support, and because seminal work in promotion of self-care skills have demonstrated its utility in understanding and predicting how behavior change takes place. [12] SCT describes an interaction between behavioral, personal, and environmental factors, all of which can be manipulated to improve a person's health and well-being. Our intervention addresses each of these three areas (Figure 2). By combining easy-to-read educational materials with one-on-one skills education and structured motivational messages we build skills (behavioral factor) and self-efficacy (personal factor) to achieve optimal health promoting behavior. Our intervention addresses environmental factors during the initial education session and the series of supportive follow-up phone calls by systematically assessing barriers and helping the patients overcome them. In addition to the SCT theory driven design, we included the notion of TTG specifically to address barriers related to knowledge acquisition. By assessing patients' knowledge and understanding, we can continue to teach until the knowledge and behavior goals are achieved.

### F1. Diuretic Self-Adjustment

**Figure 3:**
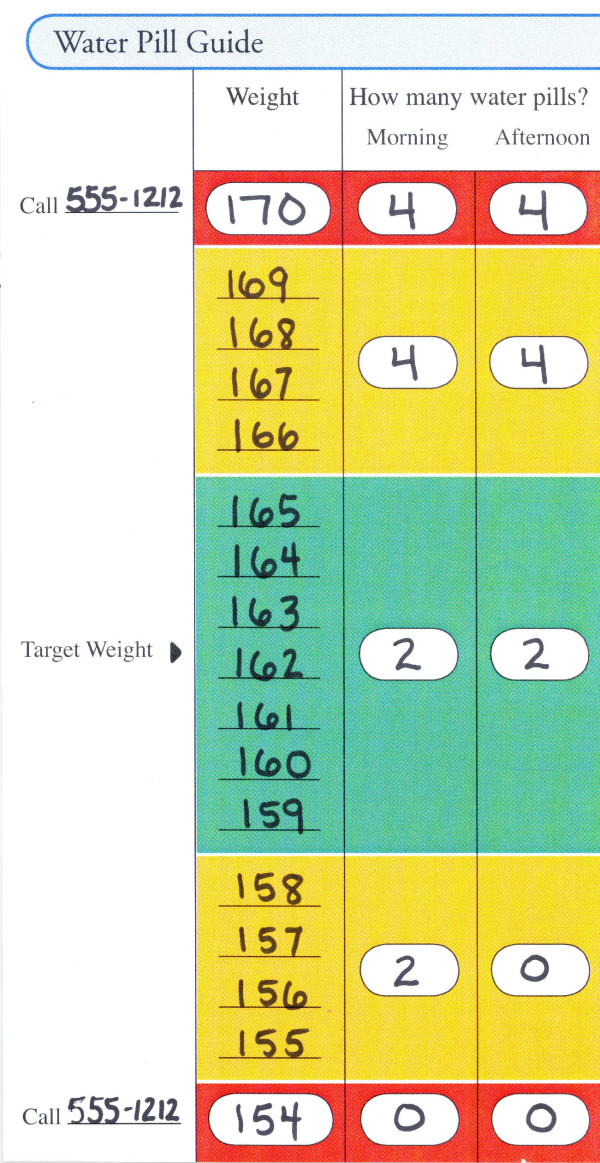
**Water pill guide**.

Only patients in the TTG group are taught to adjust their diuretic dose based on changes in their weight. A standardized algorithm was developed and tested in the pilot randomized controlled trial at UNC (Table 3). [8] The adjustment schedule taught to a patient is transcribed onto a Water Pill Guide (Figure 3) by writing the usual number of diuretic tablets he/she takes and his/her target weight (determined by the patient's physician when the patient is euvolemic) in the "green zone". From there, the rest of the Water Pill Guide is completed indicating that when the patient's weight stays within the "green zone" (3 lbs +/- of this target weight), he/she continues on their prescribed dose. If his/her weight increases or decreases by 4–7 lbs, he/she moves into the "yellow zone" and is instructed to take a different dose according to the algorithm. If his/her weight stays in the "yellow zone" for 3 consecutive days, the patient is instructed to call his/her doctor. If his/her weight deviates from the target weight by 8 pounds or more, the patient may be instructed to take yet a different diuretic dose according to the algorithm and to call his/her doctor immediately.

**Table 3:** Diuretic Adjustment Algorithm

**Instructed adjustment**	**Instructed adjustment**	**Prescribed loop diuretic dose and frequency**	**Instructed Adjustment**	**Instructed Adjustment**
**Low Red Zone**	**Low Yellow Zone**	**Green Zone**	**High Yellow Zone**	**High Red Zone**

**Weight 8# or less than target**	**If Weight is 4–7#** **Less than target**	**Target weight +/- 3 lbs** **(Euvolemic)**	**If Weight 4–7 #** **More than target**	**Weight 8# or more** **Above target**

nothing	nothing	20 mg qd	20 mg bid	20 mg bid

nothing	20 mg qd	20 mg bid	40 mg bid	40 mg bid

nothing	20 mg qd	40 mg qd	40 mg bid	40 mg bid

nothing	40 mg qd	40 mg bid	80 mg bid	80 mg bid

nothing	40 mg qd	80 mg qd	80 mg bid	80 mg bid

nothing	half dose qd	> 80 mg qd	> 80 mg bid	> 80 mg bid

nothing	80 mg qd	80 mg or more bid	80 mg bid, thiazide 25 mg qd*	80 mg bid, thiazide 25 mg qd*

nothing	160 mg qd	160 mg bid	160 mg bid, thiazide 25 mg qd*	160 mg bid, thiazide 25 mg qd*

**Alternative schedule if Physician prefers diuretic doubled in one does vs. two**				

Nothing	nothing	20 mg qd	40 mg qd	40 mg qd

Nothing	20 mg qd	40 mg qd	80 mg qd	80 mg qd

Nothing	40 mg qd	80 mg qd	160 mg qd	160 mg qd

nothing	80 mg qd	160 mg qd	160 mg bid	160 mg bid

The target weight is confirmed by the physician at time of enrollment and reflects the weight the patient is at when they are euvolemic and at baseline regarding their HF symptoms. Over the course of the year, the patient documents daily weights and diuretic dose and sends these records to the educator each month. Through calls and the monthly mailings the educator also periodically monitors daily weights along with symptoms. If the patient slowly gains or loses weight and is asymptomatic, the educator will speak to the doctor about readjusting the target weight due to the gain or loss of "dry weight". The educator will then re-set the patient's target weight, prepare a new Water Pill Guide and mail it to the patient.

Each patient's physician is allowed to customize the standard diuretic adjustment plan for an individual, but they are encouraged to accept the algorithm unless they have strong opinion that a different plan is better for their patient. In rare cases, physicians or the educator may judge that it would not be safe to allow a patient to self-adjust the diuretic medication (e.g., not reliable with the study protocol, not fully understanding the adjustment regimen, physician experience with the patient). In such cases, the patient is simply instructed to take their standard prescribed dose, monitor symptoms and to phone their physician if their weight changes such that it is no longer in the euvolemic "green zone" or if they experience symptoms. In this situation, the patient still remains in the TTG group and receives the similar degree of interaction with the educator reviewing the other core components of the training.

### F2. TTG phone call follow-up schedule

Based on our prior experience and the concept from behavior change studies, repetition and reinforcement are critical to learning new skills and integrating new behaviors into one's life. Thus our protocol includes follow-up calls that we believe are essential to transforming and solidifying self-management skills, particularly among those with low literacy whose ability to integrate new information may be more challenging. As such, we have created a standard curriculum and schedule for the calls (see Table 4).

**Table 4:** Behavior and Knowledge Requirements to Reach Goal

**Daily Self Assessment & Having a Plan**	**Call sequence**	**Call on day**
***BEHAVIOR – Verify the patient:***	Focus on during 1^st ^& 2^nd ^call,	Day 3 par & Day 7
• weighs self daily and at the correct time		
• records weight on the Daily Water Pill Plan (Figure 4)	Review on all calls	
• assess symptoms of heart failure (SOB, edema, dizziness)		
• takes the correct dose of diuretic according to their weight		
• phones doctor when appropriate		

**Medication Adherence**		

***KNOWLEDGE – Verify the patient knows:***	3^rd ^call	Day 10 (and Day 14 if need it)
• his/her fluid pill		
• What to do if they have side effects from their medications		
• That they still need to take their medications even if they feel good		
• what to do if they are unable to pay for their medications		
• how many refills they have left		
• how to get refills		
• what to do if they are out of pills		
• the importance of having a successful system for taking their medication		
• to take all their medications to their doctors appointments and reviews them with the doctor		

***Salt Avoidance***		

***KNOWLEDGE – Verify the patient knows:***	4^th ^call	Day 17 (and Day 21 if need it)
• what sodium is		
• why salt is bad for someone with heart failure		
• ways to eat less salt		
• how to tell whether something has too much salt		
• which foods are good choices based on reading nutrition labels and knowing how much sodium per serving a food has		
• which foods out of a list of 20–30 common foods are good choices		
• if foods that say "lower sodium" or "reduced sodium" are OK		
• what foods could be substitutes for high sodium foods		

**Exercise**		

***KNOWLEDGE – Verify the patient knows:***	5^th ^call	Day 24 (and Day 28 if need it)
• that it is safe for someone with heart failure to exercise		
• how exercise helps people with heart failure		
• when it is not safe to exercise		

During the first 1–2 months, the educator makes 5–8 calls to the TTG participant, each lasting about 10 minutes. During the first 2 calls, the health educator focuses on reviewing the key behavior components of the program which are performing daily weights, recording that weight, assessing for symptoms, taking the proper dose of diuretic according to their weight, and calling the doctor when appropriate. This information continues to be reviewed at every call to assess program adherence. Calls 3–8 (or beyond if needed) focus on the other three elements of HF self-management, which include medication adherence, limiting salt, and exercise. The goal of these calls is to review the content from the initial education session and then assess the patient's knowledge and behaviors regarding that content. The patient must correctly answer standard questions regarding each component on two separate occasions before the educator considers that subject as having reached the goal for that component. Once the subject has mastered the learning and behavioral goals for each component, the educator reduces the call frequency to once per month. If, at the end of the first 5–8 calls, the participant still has not achieved the learning goals, the educator will continue with calls every other week until goals are reached. This approach has been shown to improve diabetes and asthma knowledge in other studies. [13,14]

The educator records the calls in a database including the length of the calls, number of call attempts, content, whether/when the patient mastered the section or reached any goals set such as weighing themselves regularly or specific exercise goals.

As part of the behavior component, the educators also help patients identify barriers to effective care and provide motivation for adherence to the key self-care behaviors. Personal goals regarding exercise or changes in eating habits may be set by the patient and are then regularly discussed in the follow-up phone calls with the educator.

Another educational facet that is addressed with the patient is how to interact with the health care system when they need to. Patients are given specific instruction on *when *they should call their physician regarding their heart failure. Coupled with that, the patients are provided with very specific guidance on *how *to contact their physician including daytime and after-hours numbers and, in certain situations, specific steps to take to get messages to their physician. Patients are encouraged to go to appointments with all their medications, write down any questions they have for the physician and discuss those questions at the appointment.

### F3. Patient Recordkeeping Requirements

All patients enrolled in the study are taught the importance of writing down their weight daily. The patients in the TTG group are further asked to record their daily weight and daily diuretic dose on a log that is supplied to them and asked to mail them back to the educator monthly, using a self addressed stamped enveloped. These logs are then reviewed by the educator as further data on how well the patient is following the protocol.

### F4. Educator interaction with participants' physicians

The educator initially receives the following information from the physician: (1) approval for enrollment, (2) information regarding the patient's dose and frequency of loop diuretic, (3) approval of the proposed diuretic adjustment schedule, (4) approval of exercise recommendations, and (5) if the patient is euvolemic at a particular weight for purposes of setting the target weight. The educator informs the physician of the patient's intervention status after enrollment and for TTG patients, notes it in the medical chart along with the diuretic adjustment instructions the patient has received. Any further communication between the educator and the physician takes place only if the target weight needs to be re-evaluated, or if clarification is needed regarding a dosage change or other significant change that affected the patient's HF care through-out the course of the year. The educators are specifically instructed NOT to act as care managers but rather to encourage and empower the patients to contact their doctor themselves. Specific instruction in the education helps patients identify when they need to call their doctor. Only when the situation is potentially dangerous for the patient and the patient is not taking action on their own, will the educator act as mediator between the patient and the physician to ensure safety, but they do not make specific recommendations to patients.

## Outcome Assessment

### A. Primary Outcome

The main outcome of interest is rate of all-cause hospitalization and death. We chose this outcome because it is less prone to measurement error and has been the most common main outcome in previous studies assessing similar interventions. [15]

At the 6 and 12 months interviews for outcomes assessment, participants are asked about any hospitalizations that occurred since enrollment or the last assessment. When a hospitalization is reported, the survey administrator (who is blinded to intervention status) requests information about the location and date of the admission. Using these data, the RA then requests medical records from the hospital, including a copy of the admitting history and physical examination, discharge summaries, labs, cardiologic and radiological procedures, and emergency department visit notes for each reported event. In addition, we attempt to obtain events that were unreported by canvassing the site hospital and any other reported hospital during the entire enrollment period of the participant.

### B. Secondary Outcomes

We assess hospitalizations due to uncontrolled HF, all cause ED visits, and uncontrolled HF present on the ED visit as secondary outcomes. ED visits are identified in the same manner described above for hospitalization. Once the records are obtained, a clinician investigator at each site, blinded to intervention status, reviews each hospitalization and ED visit systematically and uses a study protocol and their clinical judgment to determine whether (1) uncontrolled HF was present at admission and (2) whether HF was an important contributing factor to the admission. Each question is answered on a five point scale including "definitely", "probably", "unsure" "probably not" or "definitely not". Table 5 depicts the criteria examined and the formula that guides the systematic decision for determining if uncontrolled HF was present on admission. This formula can be overridden by the reviewer's clinical judgment and other evidence in the chart. In answering the second question, if HF was a contributing factor to the admission, the reviewer uses their clinical judgment.

**Table 5:** Criteria for Uncontrolled Heart Failure

**Criteria for Uncontrolled Heart Failure**
1. Shortness of breath or edema
2. Any of the following objective indicators of cardiac dysfunction or elevated pressures
a. Newly diagnosed reduced LVEF OR
b. Elevated jugular venous pulse OR
c. Elevated BNP OR
d. CXR showing congestion or pulmonary edema
3. Received intravenous or increased oral diuretic therapy
4. Discharge diagnosis of Heart Failure (primary or secondary)

➢ Uncontrolled HF is Definitely present on admission if:
◦ All 4 criteria are present
➢ Uncontrolled HF is Probably present on admission if:
◦ Criteria 1 & 2 are present and 3 *or *4

To validate this process, we developed a re-review and adjudication procedure. Assessments are re-reviewed by a second clinical investigator if the first assessment noted worsening HF as either 'probably' or 'probably not' a contributing factor to the admission or if it is marked 'unsure'. These criteria result in approximately 40% of all events requiring re-review. The first and second reviewers' assessments are then compared. If the first and second assessments differ such that one categorized HF as a contributing factor and the second categorized it as not being a contributing factor, or if one of the reviewers is unsure, then the event is sent to the adjudication committee, made up of the three clinician-investigator outcome assessors. The adjudication committee convenes regularly by phone to review such cases. The first 2 reviewers initially discuss the case and see if a consensus can be reached. If no consensus can be reached, the third reviewer's assessment will provide the final judgment on the event.

Other secondary clinical outcomes include analysis of changes in HF-related quality of life, HF knowledge, and HF self-management behaviors. At 1 month, 6 months and 12 months, we survey patients regarding heart failure-related quality of life, mastery of knowledge regarding heart failure, self-care behavior, and self-efficacy regarding heart failure. We are using the ICICE Heart Failure Symptom Scale, which was developed for telephone interviews by adapting questions from the MLHF and other HF health status scales.[16] We will also be measuring the adoption of appropriate self management knowledge and behaviors between groups and examining if they affect the hospitalization or death rates. Lastly, we are rigorously collecting the time spent coaching the patient in the TTG intervention and will be able to assess time spent, number of calls, problems getting in touch with patients over the phone, and time it takes to master the key elements.

### C. Adverse Events

For each hospitalization or emergency department visit, we assess whether the event was caused by the study intervention, most likely but not exclusively caused by and error in the patient self titrating their diuretic. We look specifically for low potassium levels (less than 3.0 mg/dl), impaired renal function (a rise of 0.5 mg/dl or greater from last recorded value), hypovolemia, and syncope. For each event in which one of these circumstances occurred, the reviewer is unblinded to the intervention and any records from the educator are obtained to determine if there was a possible cause and effect relationship between the patient following the study protocol and the hospitalization or ED visit. In addition to this systematic review of all events, the educator is instructed to bring to the site investigator's attention potential adverse events that she learns about through her phone calls that she feels may be related to the intervention. The site team reviews these circumstances using the same criteria as listed above to determine if an adverse event related to the intervention occurred. While we recognize that this method of adverse event surveillance is biased to differently detect possible adverse events among patients that are randomized into the intervention group, we decided this protocol as a way to quickly detect any safety concerns regarding the intervention. As a final step, all study-related adverse events and all deaths are reported to the IRB at each site and to the DSMB.

### D. Sample size calculation

The sample size calculation is based on the results of our pilot trial and other self-care intervention trials. [8]In estimating the necessary sample size, we incorporated the negative binomial model into our calculations. Unlike conventional sample size calculations for continuous variables, sample size calculations for negative binomial models have no closed form solution. We estimated the power of the study for a given set of sample size and parameters by simulating data and determining the proportion of simulations for which the null hypothesis is rejected. For our preferred calculations of an incident rate ratio (IRR) of 0.7 (based on a somewhat conservative effect size from the aforementioned pilot study), we find that a sample size of 300 cases and 300 controls will yield a power of approximately 0.90. We verified our results using conventional sample size calculations and the results are similar: conventional t-test based sample size calculations yield a sample size of 253 in each arm. In order to ascertain the sensitivity of these results to alternative sample sizes and true IRRs, we repeated this experiment for other parameters; with a sample size of 300, our power drops to 0.7 if the estimated IRR is changed to 0.7.

For subgroup analysis, we are particularly interested in estimating the effect size among patients with low literacy. We are aiming for a sample of 300 patients with low literacy which will give us a power of 0.7 to identify an IRR of 0.7. If, as in the pilot study, the effect is stronger in patients with low literacy, the power will increase. For example, if the IRR is 0.6, our power will exceed 0.9.

We aim to study 600 patients with HF. We anticipated up to a 10% dropout rate based on our previous studies with similar populations. As such, we plan to recruit 660 patients for participation.

## Discussion

HF self-management support is a key element to optimal heart failure care and patients with low literacy are particularly vulnerable to poor uptake of complicated self-management training. Our pilot randomized controlled trial demonstrated the benefit of this approach for reducing hospitalizations and demonstrated that it was safe and effective in patients with low literacy. This new trial seeks to extend those findings to a multi-site study. Now we are asking in a large multi-center trial whether a one time session can be as effective as an intensive teach to goal approach to maximize understanding and behaviors. Moreover, we have powered this study to evaluate the effect of the intervention in patients with low literacy and higher literacy separately. The results of this study will help to inform the principles of design of self-management support interventions for chronic illnesses.

This study is designed to evaluate the incremental difference of an ongoing educational program compared to a one-time session. In a previous study of patients with diabetes, those who received ongoing enhanced care beyond the one time session had substantially better glycemic control. [17] In our pilot randomized control trial of HF self-management support, we found that, while most patients needed ongoing support and encouragement to master the behavioral skills for optimal self-management, this was particularly true for those with low literacy. [18] Additionally, several studies in a variety of contexts have shown that one-time didactic educational sessions are ineffective for improving health outcomes [19]

For this study, we hypothesized that ongoing education until knowledge and behavioral goals are met will benefit all patients, but particularly those with low literacy skills. Self-management of heart failure requires knowledge and application of several tasks including assessment and response to symptoms and weights. Patients with proficient literacy and numeracy skills may learn these tasks faster and more reliably than those with low literacy, particularly in the current care system, in which self-management support is haphazard. Organized, better directed care could overcome such disparities. [20] As such, we will evaluate subgroups of low and high literacy to see if the benefits are greater for the lower literacy group.

Some recent studies of disease management for HF and other conditions have shown minimal effectiveness, but most of these studies enrolled patients at lower risk, had low intensity structure, and did not necessarily seek out and enroll only those who are most in need of these services.[21,22] Low literacy, as a barrier to effective self-management, may be an important target for population disease management. Sisk and colleagues tested a nurse management intervention among socioeconomically vulnerable patients and found improved functioning and fewer hospitalizations for those who received the intervention. [23] Literacy was not an important predictor of response to the intervention in that study; patients with both low and higher literacy benefited. [24] In contrast, DeBusk and colleagues found that a nurse intervention among lower risk patients was not successful in reducing hospitalization. [21] Appropriately designed disease management focused on teaching self-management may be more effective in populations with low literacy that often require more intensive support for mastery of the knowledge and behaviors. [20]

This study specifically excludes elements of disease management that go beyond self-management support. For example, the educator is often in the position where she/he would like to advocate for the patient and contact the physician, or follow a clinical protocol to titrate medications. [25] However, we have avoided this (unless urgency requires it) and focus on empowering the patient to contact the physician. Our hope is to build patient navigation skills rather than serve as an intermediary or patient navigator. This approach has the advantage of focusing the evaluation on the self-management aspects, but the intervention could be less effective than one that has more comprehensive case management and direct medical care, particularly if the system is dysfunctional.

In summary, this study will test whether an ongoing teach to goal self-management educational program is superior to a one-time educational session for reducing hospitalizations or death from heart failure. This study will also evaluate the relative effects of the intervention on patients with low literacy and higher literacy. The results of this study will assist in the optimal design of heart failure care.

This study is planned to take place for 5 years with enrollment ending after 3.5 years. Analysis and publication of the baseline data will take place at the completion of enrollment outcome at the end of 2009. Data collection should be complete by the end of 2010 and analysis of the results should begin at that time. In addition, we will analyze the data collected by the educators on the process of education for the purpose of addressing the generalization and dissemination of this training technique into the diverse health care systems.

## Competing interests

The authors declare that they have no competing interests.

## Authors' contributions

DD and MP developed the project and obtained funding along with DB and DS. DD, MP, DB, DS, BR, & KBD developed the design. DD, MP, VH, DB and DS developed the educational material. MH developed the analysis plan and randomization with input from DD and MP. DD and KB drafted the manuscript with input and final approval from the rest of the authors.

## Pre-publication history

The pre-publication history for this paper can be accessed here:


